# Personals

**Published:** 1917-11

**Authors:** 


### PERSONAL

Announcement of removal, travel, and other matters of interest are requested. Please report errors in the listing of any physician in the State and other directories, that we may co-operate with the State Society in securing a correct list.

---

Dr. R. C. Miller of Buffalo has opened an office at 314 Federal Building.

---

Dr. Dewitt C. Greene of Buffalo returned about Oct. 1 from a motor trip to the Adirondacks, Berkshires and Cape Cod.

---

Dr. Chas. W. Bethune has moved his office to 520 Franklin St. Practice limited to Urology.

---

Dr. John D. Flagg of Buffalo has moved his office to 473 Virginia St., practice limited to Laryngology.

---

Dr. M. J. Wilson of Warsaw is spending several weeks in Oklahoma.

Dr. Ward B. Manchester, Buffalo 1907, has left for military service and his brother, Dr. L. B. Manchester, Buffalo 1913, has moved from Akron to Batavia, to take his place.

Dr. Henry C. Lapp of N. Tonawanda has been appointed Medical Inspector of Public Schools succeeding Dr. E. T. Leonard.

Dr. Guy L. McCutcheon of Buffalo left for Phoenix, Ariz., Oct. 20, to spend several months.

Dr. Edgar A. Forsyth of Buffalo announces the removal of his office to 471 Virginia St.

Dr. Hiram P. Whitford of Bridgewater, N. Y., writes us a friendly letter. He was born Oct. 15, 1826—just 91 years before the date of this writing—at Cantonbury, Ct. He was graduated at the Eclectic College of Cincinnati in 1860. He has been Health Officer of Bridgewater for over 50 years and is still active, "able to go wherever I please without cane or other help," although he has handed his practice over to his son, and writes a better hand than we can, though that is not as high a compliment as it might seem. He remembers the writings of Dr. Flint, our first editor, as current events.

Dr. Moses Rosenberg of Rochester, surgeon on the British steamer Verdi, sunk by a German submarine, was among those rescued.

Dr. Charles G. Wagner, Supt. of the Binghamton State Hospital, is reported seriously ill.

